# The Diversity of Spiculated Erythrocytes

**DOI:** 10.1002/ajh.27560

**Published:** 2024-12-13

**Authors:** Barbara J. Bain

**Affiliations:** ^1^ Centre for Haematology, Department of Immunology and Inflammation St Mary's Hospital Campus of Imperial College Faculty of Medicine, St Mary's Hospital London UK

1


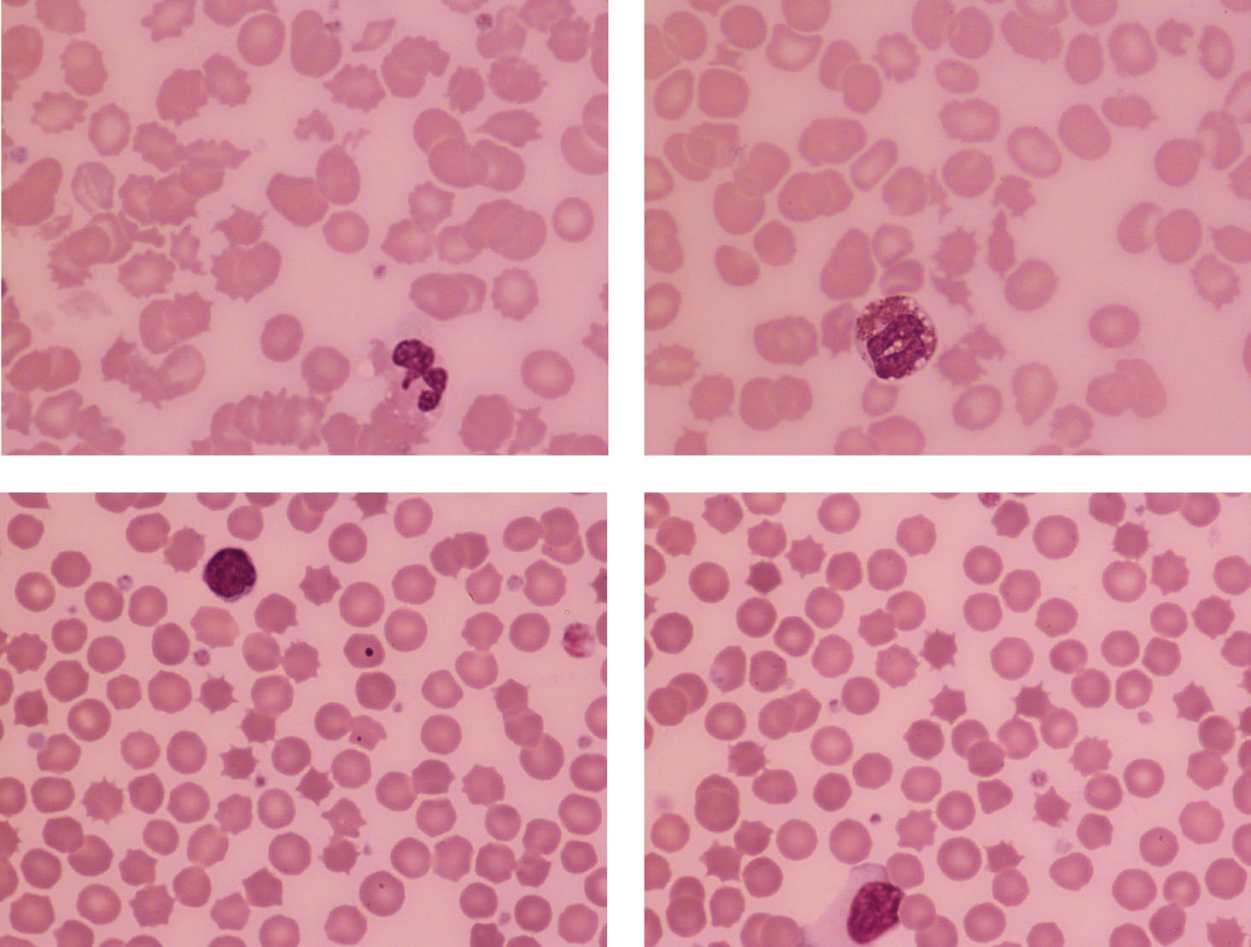
Spiculated erythrocytes come in a variety of forms, which are of diagnostic importance. They can be broadly classified as echinocytes, acanthocytes, keratocytes, and schistocytes. However, complexity is added by the superimposition of one abnormality on another. Other spiculated cells can, for example, undergo an echinocytic or acanthocytic change. In addition, other poikilocytes, for example, spherocytes, can also undergo spiculation.

An acanthocyte is an erythrocyte with a small number of irregularly shaped and irregularly disposed projections on its surface. The name comes from the Greek, *άκανθα*, meaning thorn. These cells are most often a feature of hyposplenism but also occur in liver failure, when the term “spur cell hemolytic anemia” has been used. The upper images show acanthocytosis in a patient with drug‐induced acute liver failure (all images May–Grunwald–Giemsa, ×100 objective). Acanthocytes also occur in rare inherited disorders such as the McLeod phenotype and choreo‐acanthocytosis [1]. The lower images show a more complex situation. Here we have spheroacanthocytes in a patient with hereditary spherocytosis who has had a splenectomy. The formation of the spicules has been constrained by the spherical form of the cell.

An echinocyte is a cell with numerous short regular spicules. The name comes from the Greek, *εχĩνος* meaning sea‐urchin. Echinocytosis is most often seen as a storage artifact, when it is referred to as crenation. It is a feature of pyruvate kinase deficiency, particularly post‐splenectomy when the defective cells persist in the circulation [2].

A keratocyte is a cell with two or four paired spicules, from the Greek, *κέρα*ς, for horn, while a schistocyte is a jagged red cell fragment, from the Greek, *σχίζω*, for split or divide. Both are features of microangiopathic and mechanical hemolytic anemias [3]. Keratocytes are also seen in Heinz body hemolytic anemia, as in acute hemolysis in glucose‐6‐phosphate dehydrogenase deficiency.

An appreciation of these diverse spiculated cells can be important in diagnosis.

## Conflicts of Interest

The author declares no conflicts of interest.
